# Reply to Tabeling et al. Comment on “Grabala et al. Radiological Outcomes of Magnetically Controlled Growing Rods for the Treatment of Children with Various Etiologies of Early-Onset Scoliosis—A Multicenter Study. *J. Clin. Med.* 2024, *13*, 1529”

**DOI:** 10.3390/jcm13113018

**Published:** 2024-05-21

**Authors:** Pawel Grabala, Munish C. Gupta, Daniel E. Pereira, Michal Latalski, Anna Danielewicz, Pawel Glowka, Michal Grabala

**Affiliations:** 1 Department of Pediatric Orthopedic Surgery and Traumatology, University Children’s Hospital, Medical University of Bialystok, Waszyngtona 17, 15-274 Bialystok, Poland; 2 Paley European Institute, Al. Rzeczypospolitej 1, 02-972 Warsaw, Poland; 3 Department of Orthopaedic Surgery, Washington University in St. Louis, 660 S. Euclid Ave., St. Louis, MO 63110, USA; munishgupta@wustl.edu (M.C.G.); d.e.pereira@wustl.edu (D.E.P.); 4 Paediatric Orthopaedic Department, Medical University of Lublin, Gebali 6, 20-093 Lublin, Poland; michallatalski@umlub.pl (M.L.); anna.danielewicz@umlub.pl (A.D.); 5 Department of Spine Disorders and Pediatric Orthopaedics, Poznań University of Medical Sciences, 28 Czerwca 1956 r. Street, No. 135/147, 61-545 Poznań, Poland; pawel.glowka@ump.edu.pl; 6 2nd Clinical Department of General and Gastroenterogical Surgery, The Medical University of Bialystok Clinical Hospital, Medical University of Bialystok, M. Skłodowskiej-Curie 24a, 15-276 Bialystok, Poland; michal@grabala.pl

We are immensely gratified by the considerable interest our study has garnered [1], leading to a multitude of correspondences and official remarks [2]. The scrutiny of our work and the comprehensive analysis of the research findings will result in a juxtaposition with other studies [3,4,5,6,7,8,9], thereby enriching the advancement of spinal surgery and enhancing the surgical methodologies employed in treating pediatric patients afflicted with early-onset spinal deformities. Our study’s objective was to examine the surgical interventions for early-onset scoliosis stemming from diverse causes, utilizing a methodology akin to previous investigations of magnetically controlled growing rods (MCGRs). Our focus was on meticulously evaluating the outcomes of patients subjected to the MCGR growing system. The surplus data may have caused variations in the analysis of parameters across different groups, potentially influencing the ultimate outcome [3,4,5,6,7,8,9]. We acknowledge the curiosity of researchers engrossed in diverse growing systems, as depicted by prior works, regarding the intricacies of our study. We express gratitude for the attention paid to segregating patients treated with MCGR from those who have completed the treatment and proceeded to final correction with spondylodesis. It is worth noting that our research was carefully conducted, and the amassed research findings were intended for publication across a series of publications rather than a singular one; however, due to numerous inquiries such as this letter to the editor, the remaining data will be incorporated into the subsequent response [1,2].

As elucidated by the authors [2] of the aforementioned study, the calculation for growth in the T1–T12 and T1–S1 segments entailed subtracting the initial postoperative value from the latest postoperative value [1]. This methodology was consistently applied to ascertain the maintenance of curve correction. Consequently, the amalgamation of curve values throughout the observation period of MCGR treatment and postdefinitive surgery with posterior spinal fusion (PSF) into a single group and averaging them led to a reduction in the principal curve during growth-friendly treatment. Indeed, with respect to the actual efficacy of distraction-based implants, a diminishment in correction is frequently observed, as aptly observed by the authors. They also posited that incorporating radiographs’ postfinal fusion was crucial in evaluating the performance of MCGR, potentially inflating the corrective impact of this approach, both in curve correction and growth. We respectfully disagree with this viewpoint, as the X-ray imagery is intended to reflect the authentic outcome of the treatment, exemplifying an efficacious surgical approach. It is widely acknowledged that the efficacy of a surgical technique is contingent upon its adherence to medical indications and execution by a proficient surgeon specialized in the method.

At the recommendation of the authors of the commentary [2], we have opted to disclose the actual radiological findings of patients undergoing treatment with the MCGR system, commencing from the initiation of treatment to the elongation of the MCGR rods until the conclusion of this process, prior to the ultimate surgical procedure. Table 1 comprises the radiological statistics of patients managed with MCGR, encompassing the final assessments before the conversion of MCGR to PSF, with the exception of the outcomes after the final surgery and PSF. These findings mirror the comparable accomplishments of other investigators [3,4,5,6,7,8,9,10,11,12,13,14,15]. Examination of the growth from T1–T12 and T1–S1 delineated the average growth rates for T1–T12 and T1–S1 during the treatment regimen as 5.95 mm and 10.1 mm per annum, respectively, in accordance with existing studies within the medical literature [15,16,17,18,19].

Moreover, it has been observed that with each successive year of MCGR therapy beyond 2 years, the potential for elongating T1–T12 and T1–S1 diminishes, with these values possibly decreasing by an average of up to 20% each subsequent year [19]. Furthermore, congruent T1–T12 and T1–S1 extension values were noted during MCGR insertion surgery, with T1–T12 and T1–S1 parameters escalating by 52% and 51% of the total length achieved since MCGR implantation through the elongation process, resulting in an increase in T1–T12 and T1–S1 by 48% and 49%, respectively [15]. In reference to the analysis by the authors of the commentary concerning the T1–T12 and T1–S1 values [2], and the Cobb angle of the primary curvature, it can be deduced from our findings that despite the adverse parameters and potential complications arising from MCGR treatment, the application of magnetic rods allows for the attainment of satisfactory radiological and clinical outcomes.

It is essential to acknowledge that the methodology of MCGR placement that we employ [20,21,22] incorporates minimally invasive entry (restricts spontaneous spondylodesis), along with the utilization of larger transpedicular screw dimensions (to mitigate the risk of pull-out and enhance biomechanical characteristics) [21], which may exert a notable influence on clinical and radiological outcomes and the potential for complications and subsequent interventions [13,14], thereby potentially diminishing the ultimate results for T1–T12, T1–S1, and the definitive rectification of spinal deformity. Imperfections exist in all implants; yet we anticipate that our investigations and findings, as well as those of the authors of the commentary [1,23,24,25], will contribute to substantial advancements and enhancements in the surgical management of early-onset scoliosis.

## Figures and Tables

**Table 1:** Radiological outcomes of MCGR patient cohort. FFU means final follow-up.

Variable	IS (n = 58) A	SS (n = 42) B	NS (n = 51) C	CS (n = 10) D	Total (n = 161)
Mean (SD) age at MCGR implantation in years	7.2 (2.8)	8.3 (2.3)	7.6 (3.1)	8.4 (2.2)	7.08 (2.3)
Mean (SD) FFU in months before graduation surgery	37.5 (22.5)	32.5 (23.2)	35.1 (21.8)	36.2 (22.3)	35.8 (23.2)
Mean (SD) preoperative major curve, degrees	85.3 (22)	82.5 (24)	88.2 (25)	81.9 (19)	86.2 (21)
Mean (SD) postoperative major curve, degrees	45.3 (12)	51.2 (16)	44.8 (11)	44.6 (10)	46.9 (14)
Mean (SD) major curve at FFU before graduation surgery, degrees	49.6 (10)	55.6 (9)	48.2 (8)	49.2 (10)	50.2 (11)
Mean (SD) preoperative thoracic kyphosis, degrees	55.2 (21)	39.4 (19)	35.3 (17)	47.1 (19)	47.2 (20)
Mean (SD) postoperative thoracic kyphosis, degrees	40.1 (18)	38.4 (16)	33.2 (15)	39.3 (14)	47.1 (17)
Mean (SD) max. thoracic kyphosis at FFU before graduation surgery, degrees	32.6 (8.4)	36.4 (9.2)	34.5 (8.2)	37.2 (7.8)	34.8 (8.6)
Mean (SD) preoperative lumbar lordosis, degrees	45.7 (16)	40.2 (15)	45.8 (14)	41.7 (13)	44.2 (14)
Mean (SD) postoperative lumbar lordosis, degrees	41.9 (12)	42.4 (11)	36.9 (12)	37.7 (13)	39 (12)
Mean (SD) lumbar lordosis at FFU before graduation surgery, degrees	46.5 (8.8)	37.8 (12.6)	39.4 (11.2)	40.2 (12.6)	45.8 (11.8)
Mean (SD) preoperative T1–T12 height in mm	138 (34)	158 (38)	165 (41)	142 (38)	166 (36)
Mean (SD) postoperative T1–T12 height in mm	165 (37)	183 (39)	190 (38)	171 (38)	188 (39)
Mean (SD) T1–T12 height in mm at FFU before graduation surgery	186 (39)	201 (36)	204 (35)	183 (38)	208 (36)
Preoperative vs. FFU comparisons	*p* < 0.001	*p* < 0.001	*p* < 0.001	*p* < 0.001	*p* < 0.001
Postoperative comparisons					
A vs. B *p* > 0.05	A vs. C *p* > 0.05	A vs. D *p* < 0.001	B vs. C *p* > 0.05	B vs. D *p* < 0.001	C vs. D *p* < 0.001
Mean (SD) preoperative T1–S1 height in mm	282 (58)	293 (62)	282 (65)	262 (58)	295 (65)
Mean (SD) postoperative T1–S1 height in mm	317 (55)	328 (58)	315 (58)	297 (54)	328 (63)
Mean (SD) T1–S1 height in mm at FFU before graduation surgery	348 (52)	354 (55)	340 (48)	317 (51)	360 (58)
Preoperative vs. FFU comparisons	*p* < 0.001	*p* < 0.001	*p* < 0.001	*p* < 0.001	*p* < 0.001
Postoperative comparisons					
A vs. B *p* < 0.001	A vs. C *p* < 0.001	A vs. D *p* > 0.05	B vs. C *p* > 0.05	B vs. D *p* < 0.001	C vs. D *p* > 0.05
Mean preoperative T1–T12 height obtained during initial correction in %	56%	58%	64%	70%	52%
Mean (SD) preoperative T1–T12 height obtained during FFU correction in %	44%	42%	36%	30%	48%
Mean (SD) preoperative T1–S1 height obtained during FFU correction in %	53%	57%	57%	63%	51%
Mean (SD) preoperative T1–S1 height obtained during initial correction in %	47%	43%	43%	37%	49%
Mean (SD) length of distraction phase, months	36.5 (14.2)	33.9 (9.8)	29.7 (11.2)	26.8 (8.8)	37.5 (13.8)
A vs. B *p* > 0.05	A vs. C *p* < 0.001	A vs. D *p* < 0.001	B vs. C *p* > 0.05	B vs. D *p* > 0.05	C vs. D *p* > 0.05
Mean (SD) number of lengthenings per 1 year	6.5 (1.5)	5.5 (2.2)	5.8 (1.2)	6.2 (1.4)	6.7 (1.3)
A vs. B *p* > 0.05	A vs. C *p* > 0.05	A vs. D *p* > 0.05	B vs. C *p* > 0.05	B vs. D *p* > 0.05	C vs. D *p* > 0.05
Mean (SD) T1–T12 growth mm/year at FFU	6.2 (1.8)	5.8 (1.2)	5.5 (1.5)	5.2 (1.1)	5.95 (2.2)
A vs. B *p* < 0.001	A vs. C *p* < 0.001	A vs. D *p* < 0.001	B vs. C *p* > 0.05	B vs. D *p* > 0.05	C vs. D *p* < 0.001
Mean (SD) T1–S1 growth mm/year at FFU	10.8 (3.2)	9.1 (2.8)	9.8 (2.6)	8.8 (2.8)	10.1 (3.4)
A vs. B *p* > 0.05	A vs. C *p* > 0.05	A vs. D *p* < 0.001	B vs. C *p* > 0.05	B vs. D *p* > 0.05	C vs. D *p* < 0.001
